# Engineered Genetic Circuits Activated by Bezafibrate Improve ESC‐Based TAA Cancer Vaccine Efficacy and PD‐L1 Nanobody Therapy

**DOI:** 10.1002/advs.202500272

**Published:** 2025-04-17

**Authors:** Meiling Jin, Shuzhen Liu, Mingshuo Zhan, Jian‐Dong Huang

**Affiliations:** ^1^ Chinese Academy of Sciences (CAS) Key Laboratory of Quantitative Engineering Biology Shenzhen Institute of Synthetic Biology Shenzhen Institutes of Advanced Technology Chinese Academy of Sciences Shenzhen 518055 P. R. China; ^2^ School of Biomedical Sciences Li Ka Shing Faculty of Medicine The University of Hong Kong Pokfulam Hong Kong SAR P. R. China; ^3^ Department of Clinical Oncology Shenzhen Key Laboratory for cancer metastasis and personalized therapy The University of Hong Kong‐Shenzhen Hospital Shenzhen 518053 P. R. China; ^4^ Guangdong‐Hong Kong Joint Laboratory for RNA Medicine Sun Yat‐Sen University Guangzhou 510120 P. R. China; ^5^ Materials Innovation Institute for Life Sciences and Energy (MILES) HKU‐SIRI Shenzhen 518057 P. R. China

**Keywords:** bezafibrate, cancer, embryonic stem cell, epitopes, Gal4‐UAS, immunotherapy, synthetic genetic circle

## Abstract

Immunotherapy targeting tumor antigens and immune checkpoint inhibitors has garnered significant attention in cancer treatment. Synthetic gene circuits are developed, encoded in plasmids, which regulate the expression of tumor antigens shared with embryonic stem cells (ESCs) and PD‐L1 nanobody (PD‐L1 nb) in response to bezafibrate stimulation. This approach significantly minimizes side effects and improved therapeutic efficacy. The transcriptional switches leverage the interaction between the bezafibrate‐responsive transcriptional activator PPARγ and RXRα, which are fused with the VPR/VP64/p65 activation domains (AD) and the Gal4 DNA‐binding domain (DBD), respectively. These synthetic constructs are validated and their ability to modulate gene expression upon bezafibrate treatment are confirmed. Notably, the gene expression is precise and tunable in response to bezafibrate administration. HEK293T cells or ESCs are employed to deliver this gene circuit, or the plasmids containing the circuit into the tumor are directly injected. Administration of bezafibrate reduces tumor growth, increases specific CD8^+^ T cells, and mitigates CD8^+^ T cell exhaustion, underscoring the feasibility and effectiveness of the approach. ESC‐based and intratumoral delivery of the synthetic gene circuits and cargo genes, particularly PD‐L1 nb, significantly inhibit tumor growth. PD‐L1 nb effectively blocks PD‐L1 expression both in vitro and in vivo, as confirmed by using a mutant PD‐L1 nb sequence.

## Graphical abstract

Synthetic gene circuits regulated by bezafibrate are developed to express tumor antigens and PD‐L1 nanobody (PD‐L1nb), enhancing cancer immunotherapy. Delivered via HEK293T cells, ESCs, or directly injected the plasmids containing the circuit into the tumor, the circuits reduce tumor growth, boost CD8+ T cells, and minimize T cell exhaustion. PD‐L1nb effectively blocks PD‐L1, demonstrating precise, tunable gene expression and therapeutic potential.

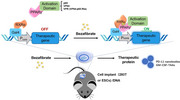

## 1 Introduction

Tumor immunotherapy has emerged as a promising approach for eliminating malignant tumors.^[^ ^1^ ^]^ Therapeutic cancer vaccines aim to stimulate the patient's adaptive immune system against specific tumor antigens to control tumor growth. However, the failure of cancer vaccines is often attributed to the presence of fewer immunogenic antigens and immunosuppressive microenvironments.^[^ ^2^ ^]^ The choice of combination therapy can significantly influence the therapeutic outcome.

Synthetic biology offers a novel approach to enhancing the immunogenicity of tumor antigens when combined with immune therapeutic methods, thereby improving tumor treatment. Synthetic genetic circuits regulated by small molecules in mammalian transgene‐control devices share a common design principle that ensures high‐level, specific, and safe transcription for gene‐ and cell‐based therapies.

For tumor therapy, we selected tumor antigens derived from ESCs, which have been successfully used to identify and inhibit the growth of bladder and lung cancers.^[^ ^3^ ^]^ However, peptide‐based vaccines face challenges such as the short half‐life of peptides and the limited duration of antitumor immune responses in vivo.^[^ ^4^ ^]^ Cytotoxic T lymphocytes (CD8+ CTLs) are crucial in antitumor immunity, but they can become exhausted upon the expression of PD‐1.^[^ ^5^ ^]^ PD‐1/PD‐L1 blockade therapy offers substantial advantages over conventional chemotherapies by mitigating T‐cell exhaustion and enhancing their cytotoxic capabilities.^[^ ^6^ ^]^ As a potential therapeutic agent, a PD‐L1 nanobody derived from camelids was selected from the Research Collaboratory for Structural Bioinformatics Protein Data Bank (RCSB PDB). Engineered probiotic bacteria expressing and secreting the PD‐L1 nb and a cytotoxic T lymphocyte‐associated protein‐4 (CTLA‐4) nanobody have been shown to reduce tumor growth and enhance T cell activation when administered intratumorally.^[^ ^7^ ^]^ Compared to traditional antibodies, which are ≈150 kDa, nanobodies are much smaller (≈15 kDa) and lack an Fc region.^[^ ^8^ ^]^ This unique structure allows nanobodies to be recombinantly produced or genetically expressed in mammalian cells.

In this study, we selected the clinical drug bezafibrate to regulate synthetic gene circuits. Bezafibrate, a potent agonist of Peroxisome proliferator‐activated receptors (PPARs, including PPARγ and PPARα), exhibits synergy with anti‐PD‐1 antibodies, enhancing the antitumor response of cytotoxic T cells ^[^ ^9^ ^]^ by promoting fatty acid oxidation (FAO) and increasing mitochondrial respiratory capacity.^[^ ^10^ ^]^ Specifically, the initial activation of CD4^+^ and CD8^+^ T cells involves modulation of mitochondrial activation and FAO, mediated by PPARγ and PPARα signaling pathways, respectively.^[^ ^9^, ^11^ ^]^ Increasing FAO through bezafibrate has been shown to expand the memory T‐cell population, suggesting that targeting the PPAR signaling pathway could effectively enhance tumor therapeutic outcomes.

The Gal4/UAS system is a versatile gene expression regulatory system initially discovered in yeast (*Saccharomyces cerevisiae*) and subsequently adapted for use in various organisms, including *Drosophila melanogaster*, mice, and zebrafish. Gal4 is a transcription factor that comprises a DBD and a transcription AD, which enables it to bind to a specific sequence known as the UAS (upstream activation sequence).^[^ ^12^ ^]^ This binding activates transcription by a basal promoter located downstream of the UAS. The Gal4/UAS system has several key advantages. First, the UAS element drives the expression of downstream genes at significantly higher levels than endogenous tissue‐specific promoters. Second, expression vectors or transgenic animals containing Gal4 and UAS constructs can be adapted and utilized across various research models. ^[^ ^12^, ^13^ ^]^

Given the advantages of the Gal4/UAS system, we have developed a novel synthetic biology‐based tumor vaccine that incorporates a synthetic circuit and therapeutic target genes. One component of this system is an inducible mechanism using bezafibrate to regulate the interaction between the RXR Ligand Binding Domain (LBD) and PPAR LBD. These domains are fused with the Gal4 DBD and the p65/VPR/VP64 transcription AD, respectively. The second component comprises therapeutic genes, including immune mediators, tumor antigens, and PD‐L1 nanobodies. We have integrated these two components and evaluated the anti‐tumor efficacy of the synthetic tumor vaccines in vivo, utilizing various delivery systems.

## 2 Results

### 2.1 Bezafibrate Regulates PPAR‐RXRα Binding to its Cognate DNA Operator Site PPRE

**Figure 1:**
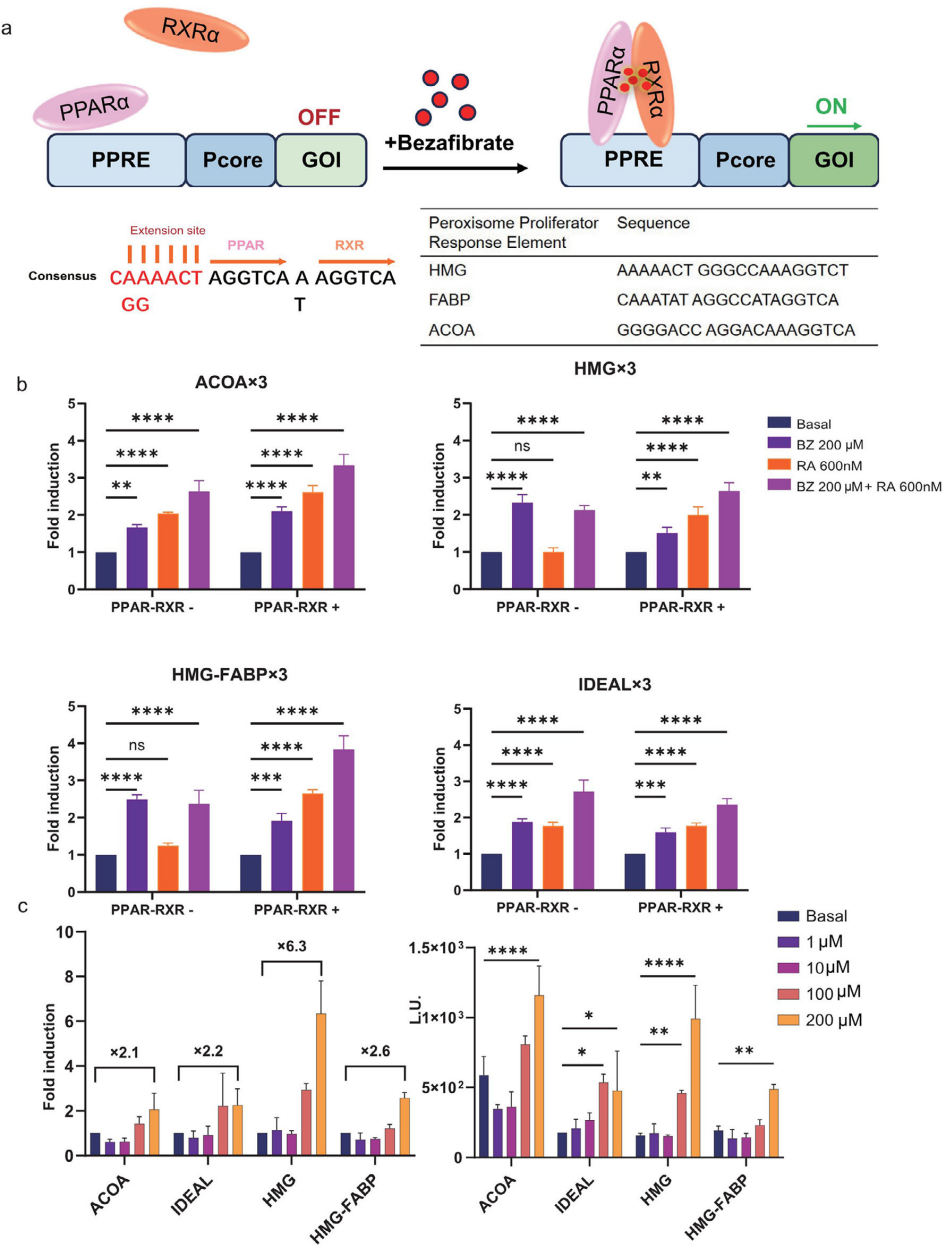
Bezafibrate‐induced gene expression through PPAR‐RXR binding to PPRE. a) Schematic illustration of the interaction between PPARα and RXRα, showing their binding to PPRE in the presence of bezafibrate. In this study, AGGTCA was chosen as the ideal PPRE sequence, while the PPRE sequences on the promoters of HMG, FABP, and ACOA were selected for subsequent analysis. b) Fold‐increase of luciferase activity (bezafibrate, retinoic acid, or together), and validation of bezafibrate or retinoic acid regulation of the pGL3‐IDEAL/ACOA/HMG/HMG‐FABP‐luciferase constructs in transiently transfected HEK293T cells. c) Fold‐increase of luciferase activity (bezafibrate), and validation of bezafibrate‐dependent regulation of the pGL3‐IDEAL/ACOA/HMG/HMG‐FABP‐luciferase constructs in transiently transfected HEK293T cells. The data represent mean values ± standard deviation (SD) (n = 3) from three independent experiments; Each experiment consisted of three replicates. Data are shown as mean ± SD. *p* values are from two‐way ANOVA followed by Tukey's post hoc test (b, c). *, *p <* *0.05*; **, *p <* *0.01*; ***, *p <* *0.001*; ****, *p <* *0.0001*.

Bezafibrate binds to the PPAR LBD and regulates PPAR by promoting the formation of a heterodimer with the Retinoid X Receptor (RXR). This heterodimer specifically binds to DNA sequences known as PPAR response elements (PPREs).^[^ ^14^ ^]^ PPREs consist of a direct repeat of the consensus hexanucleotide sequence AGG(A/T)CA, separated by a single nucleotide.^[^ ^15^ ^]^ We developed a bezafibrate‐regulated gene expression system with ON‐type behavior in mammalian cells by leveraging the interaction between PPAR and RXR with PPRE (**Figure** **1**a). To optimize this system, we selected an ideal hexanucleotide sequence (IDEAL) and identified three natural PPRE sequences within the promoter regions of genes involved in lipid metabolism: 3‐Hydroxy‐3‐methyl‐glutaryl‐CoA‐synthase (HMG), Fatty Acid Binding Protein (FABP), and Acyl‐CoA Oxidase (ACOA). These sequences have been reported to demonstrate high binding efficiency for the heterodimerization of PPAR and RXRα^[^ ^16^ ^]^(Figure 1a). By incorporating these elements into our synthetic biology‐based tumor vaccine, we aim to create a highly responsive and regulated system to enhance the expression of therapeutic genes, including immune mediators, tumor antigens, and PD‐L1 nanobodies, ultimately improving anti‐tumor efficacy in vivo.

To integrate a synthetic transcription unit into this bezafibrate‐triggered, PPRE‐dependent signaling pathway, we constructed a hybrid transcription factor, PPAR‐RXRα, incorporating DNA binding domains specific to PPREs. The promoter activity of ACOA, HMG, HMG‐FABP, and IDEAL was evaluated in the presence or absence of PPAR‐RXRα, bezafibrate, or 9‐cis‐retinoic acid, either individually or in combination, at low activation levels (Figure 1b). Consistent with previous studies, we observed that a single ligand specific to either PPARs or RXR alone can facilitate their heterodimerization and activation.^[^ ^17^ ^]^ To gain more precise insights, we further evaluated the impact of varying concentrations of bezafibrate on the binding activity of PPRE. Our results indicated that bezafibrate enhanced the promoter activity of ACOA, HMG, HMG‐FABP, and IDEAL in a dose‐dependent manner. However, this enhancement remained relatively modest for these promoters (Figure 1c). These findings suggest that while bezafibrate can activate the bezafibrate‐triggered, PPRE‐dependent signaling pathway, the level of activation may need to be optimized further for robust therapeutic gene expression in the context of our synthetic biology‐based tumor vaccine.

### 2.2 Design and Optimize the Bezafibrate‐ON Type Circuit Transcription

We hypothesized that the intrinsic properties of PPAR‐RXR, particularly their DBD affinity and ADstrength, limit their capacity to recruit transcriptional molecules, thereby constraining the performance of the bezafibrate‐ON system. Drawing inspiration from data‐driven machine learning literature,^[^ ^18^ ^]^ we analyzed three critical features: activation domain strength, promoter strength, and UAS quantity to achieve a tunable system.

**Figure 2:**
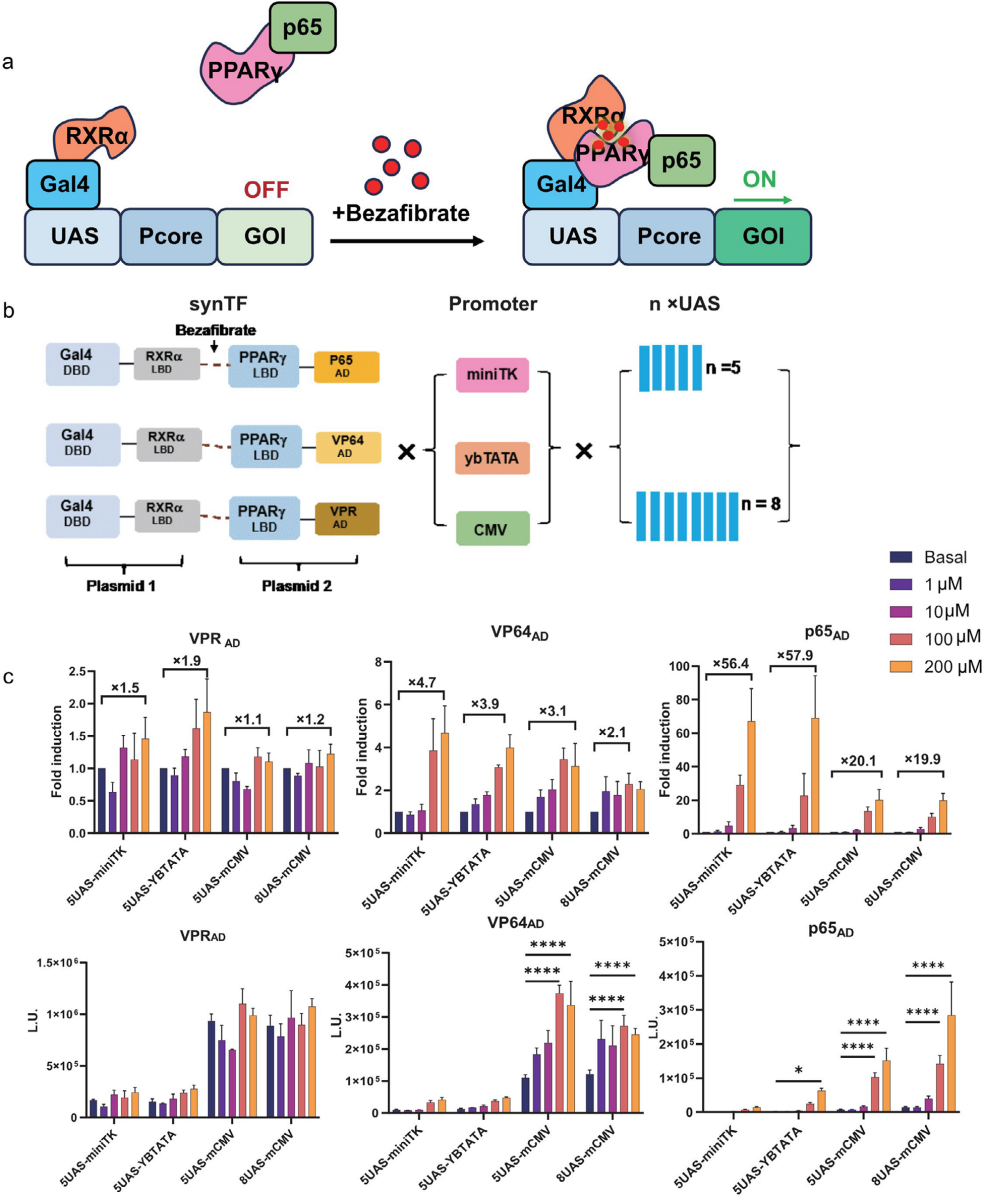
Bezafibrate‐induced gene expression control with the synthetic gene circuit Gal 4‐RXRα/PPARγ‐VPR/VP64/p65 AD‐UAS. a) Schematic illustration Gal4 DBD fused with RXRα LBD, PPARγ LBD fused with VPR/VP64/p65 AD, bezafibrate regulates the two separate part interaction and binding to UAS region to initiate downstream genes expression. b) The reporter construct used in this experiment consisted of Gal4 DBD‐RXRα LBD, PPARγ LBP‐VPR/VP64/p65 AD, miniTK, ybTATA, CMV, 5 x UAS, 8 x UAS, c) Fold‐increase of luciferase activity (bezafibrate), and validation of bezafibrate‐dependent regulation of the pcDNA 3.1 Gal4 DBD‐RXRα LBD, PPARγ LBP‐VPR/VP64/p65 AD, 5 x UAS‐P_miniTK/ ybTATA/CMV_‐luciferase, 8 x UAS‐P_CMV_‐luciferase constructs for 24 h in transiently transfected HEK293T cells. Data are shown as mean ± SD. *p* values are from two‐way ANOVA followed by Tukey's post hoc test (c). *, p < 0.05; **, p < 0.01; ***, p < 0.001; ****, p < *0.0001*.

To enhance the system, we designed a new circuit integrating the Gal4 DBD fused with RXR LBD and PPARγ LBD, coupled with three distinct activating domains (VPR, p65, and VP64). Additionally, we refined the synthetic transcription factor (synTF) by evaluating various promoter combinations (miniTK, YBTATA, CMV) and adjusting the number of UAS sequences (5, 8) (**Figure** **2**b). Upon bezafibrate‐induced interaction between PPARγ and RXRα, the Gal4‐DBD formed complexes with the respective ADs, triggering UAS‐mediated target gene expression (Figure 2a).

Luciferase assays demonstrated that constructs with the p65 AD exhibited the strongest regulatory effects, showing the highest fold change in response to varying bezafibrate concentrations, followed by VP64 and VPR, which showed the weakest performance (Figure 2c; Figure S1a‐d, Supporting Information). Interestingly, VPR AD exhibited the highest fluorescence intensity, which aligns with previous predictions (Figure 2c; Figure S1a–d, Supporting Information).

For promoter efficiency, miniTK displayed the weakest performance, followed by YBTATA, while CMV demonstrated the strongest potency (Figure 2c; Figure S1a–d, Supporting Information). Moreover, increasing the number of UAS elements enhanced the expression of the target genes, particularly when coupled with the p65 AD. Therefore, considering the efficacy of the drug as a switch to activate downstream expression and the desired intensity of the target gene expression, the p65 AD‐8UAS‐CMV construct emerged as the optimal choice. This optimized circuit was subsequently used as the therapeutic gene circuit in our experiments. By integrating these optimizations, we developed a highly responsive and effective bezafibrate‐ON type circuit, which was employed in the subsequent experiments to evaluate the therapeutic potential of the synthetic biology‐based tumor vaccine.

### 2.3 Bezafibrate‐Inducible Therapeutic Gene Expression

**Figure 3:**
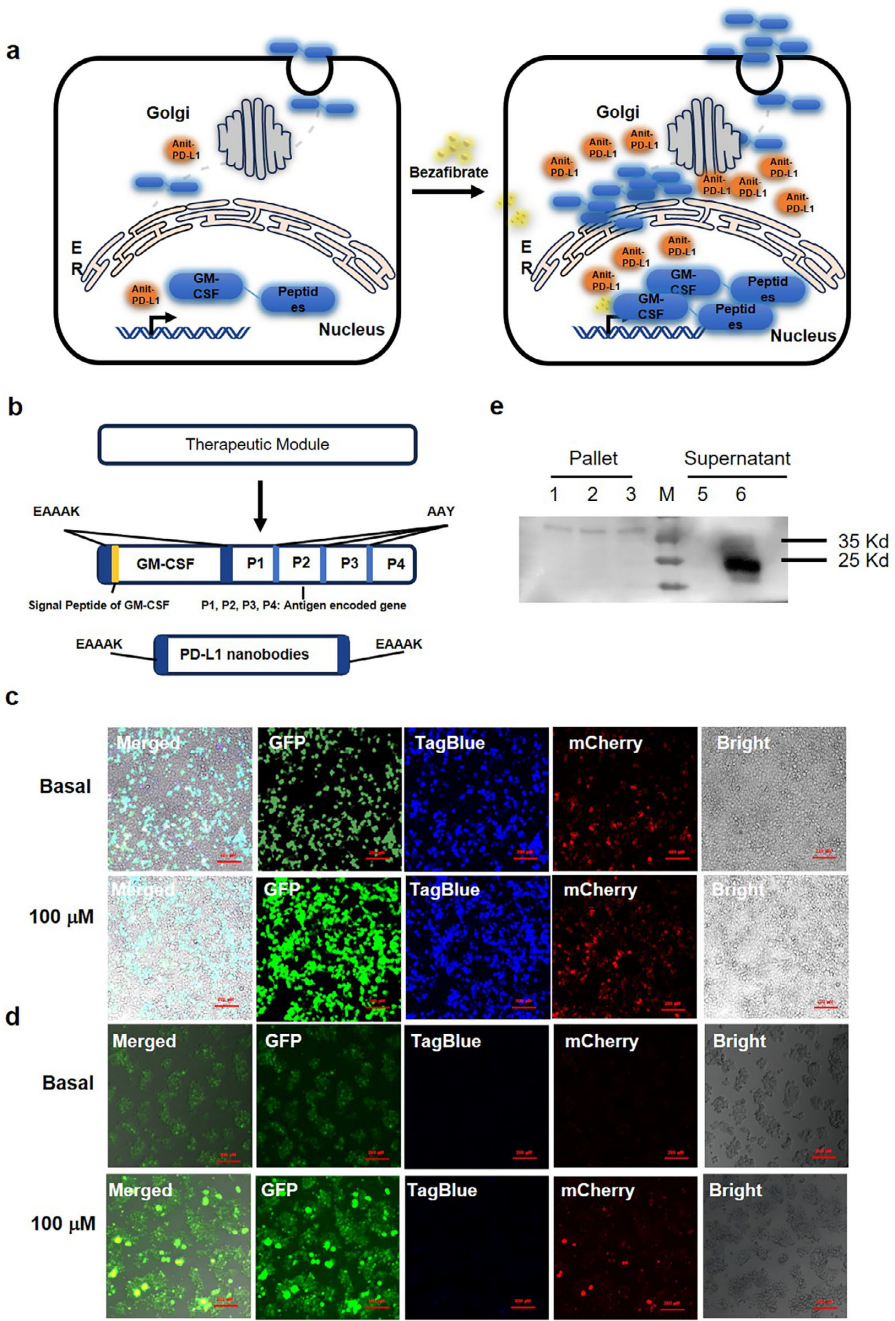
Validation of the bezafibrate‐inducible gene circuit for regulating transgene expression in cells. a) Schematic illustration of cells engineered for bezafibrate‐regulated transgene (GM‐CSF‐peptides or PD‐L1 nb) expression. b) Plasmid DNA construct encoding the bezafibrate‐regulated secretion of GM‐CSF‐peptides and the expression of PD‐L1nb. c,d) GFP‐specific fluorescence micrographs of HEK‐293T cells (c) or ESCs (d) co‐transfected with (P_Gal4‐RXR,_P_PPARγ‐p65_, P_8UAS‐CMV_) for 48 h in the presence or absence of 100 µ m bezafibrate. e) Western blot analysis of GM‐CSF secretion in the culture medium, regulated by bezafibrate. 1, 2, 3 indicate intracellular components, while lanes 5 and 6 represent the supernatant of cells transfected with P_8‐UAS_‐miniTK and P_8‐UAS_‐CMV, 5,6 treated with 100 µm bezafibrate, respectively.

The target gene expression design utilized effective immunogenic therapeutic antigen epitopes we previously screened from ESCs,^[^ ^3^ ^]^ including nuclear localized factor 2 (NUF2), claudin 6 (CLDN6), anillin (ANLN), and cyclin B (CCNB), interspersed with AAY linkers. Granulocyte‐Macrophage Colony‐Stimulating Factor (GM‐CSF), an efficacious immune therapeutic mediator, is limited by its short half‐life and high cost. ^[^ ^19^ ^]^ Consequently, in our system, GM‐CSF was incorporated and expressed in tandem with these antigenic peptides, further separated by EAAAK linkers (**Figure** **3**b). Following expression, GM‐CSF facilitates the secretion of these peptides from the cells (Figure 3a).

In the absence of bezafibrate, the target gene, regulated by the CMV promoter, exhibits minimal expression. However, its expression is significantly upregulated in the presence of bezafibrate (Figure 3a). Predictions generated by AlphaFold3 software suggest that the integrity of GM‐CSF with multiple epitope peptides preserves its biological activity (Figure S2, Supporting Information). Unlike GM‐CSF, PD‐L1 nb is exclusively expressed intracellularly due to the absence of signal peptides, thereby enhancing their safety profile within targeted tissues (Figure 3a). These designs ensure that the bezafibrate‐inducible system effectively regulates the expression of therapeutic genes, enhancing the secretion of antigenic peptides and maintaining the biological activity of GM‐CSF. The intracellular expression of PD‐L1 nb ensures a targeted and safe therapeutic approach, making this system a promising strategy for tumor immunotherapy.

Further experiments confirmed the secretion of GM‐CSF expressed in HEK 293T cells, which occurred at substantial levels. Notably, the secreted GM‐CSF displayed a higher molecular weight compared to its standard form, indicating the co‐secretion of antigenic peptides (Figure 3e). Additionally, cellular fluorescence assays demonstrated that bezafibrate effectively acts as a switch regulating the expression of the target genes. Specifically, 100 µm bezafibrate, determined as the optimal concentration, significantly enhanced the expression of the target gene within 48 hours in both HEK 293T cells and ESCs (Figure 3c,d).

Collectively, these results provide strong evidence for the regulation of therapeutic molecule expression via a bezafibrate‐controlled synthetic gene circuit. This indicates that the system is a promising strategy for cancer therapy.

### 2.4 Anti‐Tumor Effects of GM‐CSF‐Peptides Regulated by Bezafibrate

**Figure 4:**
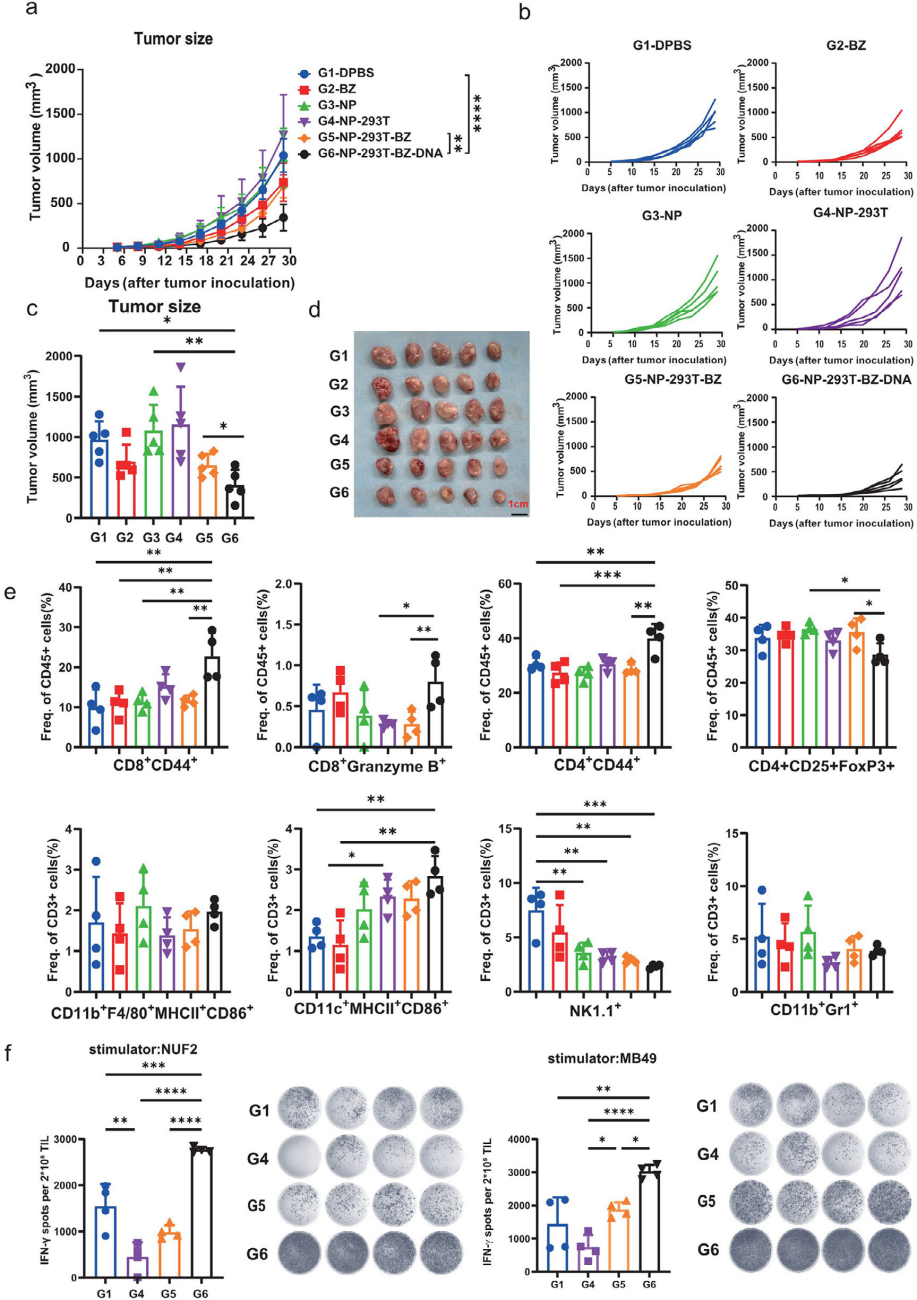
Bezafibrate‐triggered GM‐CSF and peptides expression in a mouse model of bladder cancer. a) C57BL/6J mice were s.c. injected with HEK 293 cells encapsulated in sodium alginate into the right flank on day 0, followed by s.c immunized on day 7, 14 for three total vaccinations. Bezafibrate (10mg kg^−1^) was administrated i.p. every three days. Anti‐tumor effects of bezafibrate‐triggered target gene expression were assessed (n = 5 per group). Tumor size was measured every 3 days. c,d) Tumor size on day 30 and representative images of tumors. e) Quantitative analysis of different subsets of CD3^+^CD45^+^ T cells in lymph nodes by flow cytometry (n = 4). f) Quantitative analysis of ELISPOT assay for IFNγ secretion to detect immune cell activation of TILs against NUF2 and MB49 cancer cells. Groups: G1, PBS; G2, Bezafibrate alone; G3, sodium alginate particles; G4, HEK293T cells encapsulated in sodium alginate; G5, HEK293T cells encapsulated in sodium alginate with i.p. bezafibrate administration; G6, Synthetic gene circuit expressed in HEK293T cells were encapsulated in sodium alginate and injected subcutaneously (s.c), and administered with i.p. bezafibrate. Data are shown as mean ± SD. *p* values are from one‐way ANOVA or two‐way ANOVA followed by Tukey's post hoc test or unpaired two‐tailed Student's t‐test. *, p < 0.05; **, p < 0.01; ***, p < 0.001; ****, p < *0.0001*.

To evaluate the antitumor efficacy of our optimized bezafibrate‐regulated genetic switch in vivo, we encapsulated engineered cells in alginate and implanted them in mice, which were then administered bezafibrate via intraperitoneal injection. The cells implant harboring a functional switch showed significantly reduced MB49 bladder cancer growth compared to the cells that did not implant transgenes (**Figure** **4**a–d). However, bezafibrate alone exhibited a modest inhibitory effect, whereas sodium alginate‐encapsulated HEK 293T cells potentially had some pro‐tumorigenic properties. Mice were sacrificed and draining lymph nodes (dLNs) were collected to measure the ratio of different subsets of immune cells. The GM‐CSF and peptides resulted in elevated levels of CD8^+^ effector T cells, CD4^+^ memory T cells, CD8^+^ memory T cells, and dendritic cells (DCs), whereas it had no significant effect on regulatory T cells (Tregs), macrophages, and myeloid‐derived suppressor cells (MDSCs) in the mouse MB49 model (Figure 4e). Additionally, we detected reduced levels of natural killer (NK) cells in the dLN of the GM‐CSF and peptides group (Figure 4e). Mice spleens were harvested and stimulated with synthesized peptide (NUF2) and MB49 cells. The ELISPOT assay detected more IFN‐γ‐positive spots in the spleens of the GM‐CSF‐peptides group, indicating increased activation of T cells (Figure 4f). These results demonstrate that the expression of GM‐CSF‐peptides regulated by the synthetic gene switch elicits strong and specific anti‐tumor responses, thereby reducing tumor growth.

### 2.5 Synthetic Genetic Circuit Enhanced the Anti‐Tumor Effects of ESC‐Based Cancer Vaccine

Next, we tested whether the Gal4‐RXR/PPAR‐p65‐UAS system could be applied in ESCs for tumor vaccine development. Our preliminary data have demonstrated that ESCs and tumor antigen epitopes derived from ESCs exhibit strong anti‐tumor effects.^[^ ^3^ ^]^ However, in therapeutic vaccines, adjuvants and immunomodulatory factors are still required to mitigate the suppressive effects within the immune microenvironment and elicit more potent anti‐tumor activity. Bezafibrate has shown enhanced anti‐tumor effects when modulating immune cell metabolism and in combination with PD‐1 antibodies. ^[^ ^20^ ^]^ ESC vaccines, when combined with ESC‐derived peptides, can improve the anti‐tumor effects of ESC vaccines (Figure S3b, Supporting Information). Therefore, we transfected this synthetic circuit into ESCs to regulate the expression of PD‐L1 nb or GM‐CSF‐peptides, aiming to augment its antitumor potential and enable it to function as a more potent therapeutic tumor vaccine.

**Figure 5:**
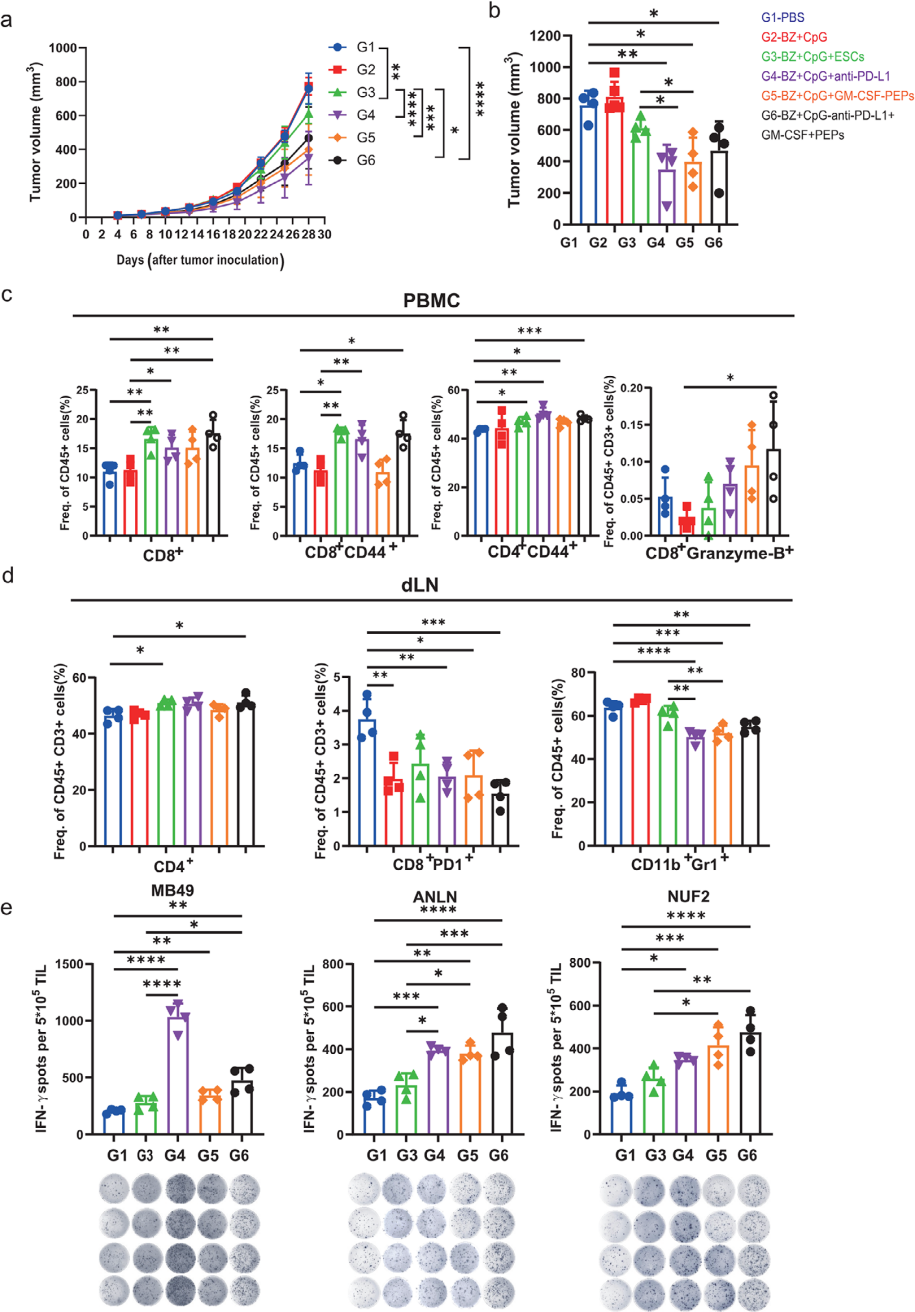
Bezafibrate‐induced expression of GM‐CSF‐peptides and PD‐L1 nb in ESCs in a mouse model of bladder cancer. C57BL/6 J mice were s.c. injected with MB49 cells in the right flank on day 0 and s.c. immunized on day 7, 14 and 21, with a total of three vaccinations (n = 4 per group). a,b) Antitumor effects observed following the expression of a synthetic gene circuit in ESC, regulated by bezafibrate. Tumor size was measured every 3 days. c,d) Quantitative analysis of different subsets of immune cells in PBMCs c) and lymph nodes d) by flow cytometry (n = 4). e) Quantitative assessment of IFNγ secretion using ELISPOT assays to detect immune cell activation in TILs against selected epitopes and MB49 tumor cells. G1, PBS. G2, Bezafibrate + CpG, G3, Bezafibrate + CpG + ESCs, G4, Bezafibrate + CpG + ESC (PD‐L1 nb), G5, Bezafibrate + CpG + ESC (GM‐CSF‐peptides), G6, Bezafibrate + CpG + ESC (PD‐L1 nb, GM‐CSF‐peptides), (n = 4). Data are presented as mean ± SD. Statistical significance was determined using one‐way ANOVA or two‐way ANOVA followed by Tukey's post hoc test or an unpaired two‐tailed Student's t‐test. *, p < 0.05; **, p < 0.01; ***, p < 0.001; ****, p < *0.0001*.

We tested the antitumor effects of this engineered ESC vaccine on the MB49 bladder cancer mouse model. The results showed that the tumor inhibitory efficacy of ESCs transfected with PD‐L1 nb, GM‐CSF‐peptides was significantly enhanced compared to that of ESCs alone (**Figure** **5**a,b). However, the combined transfection of both constructs did not yield better outcomes than single‐construct transfection, potentially due to competitive interactions occurring during the co‐transfection process (Figure 5a,b).

The mice were sacrificed, and blood samples and dLNs were collected to analyze the ratios of various immune cell subsets. The results revealed an increase in CD8^+^ cells and CD8^+^ memory cells in the ESC group, the ESCs transfected with PD‐L1 nb group, and the ESCs co‐transfected with PD‐L1 nb and GM‐CSF‐peptides group in the blood (Figure 5c). Notably, CD4^+^ memory cells were specifically elevated in the PD‐L1nb‐transfected ESC group, while active CD8^+^ T cells were augmented in the co‐transfected group in the blood (Figure 5c). Additionally, CD4^+^ T cells increased in both the ESC and co‐transfected ESC groups in dLNs (Figure 5d). Conversely, exhausted T cells and MDSCs were significantly reduced in all three engineered ESC groups in dLNs (Figure 5d). Additionally, specific T cells were increased in ESCs transfected with PD‐L1 nb, GM‐CSF‐peptides, and co‐transfected groups when stimulated with ANLN, NUF2, and MB49 (Figure 5e). These findings imply that ESCs transfected with PD‐L1 nb or GM‐CSF‐peptides positively impact effector and memory T cell expansion while decreasing exhausted T cells and MDSCs, thereby improving the anti‐tumor effects of ESC‐based vaccines.

### 2.6 Tumor In Situ Expression of Therapeutic Genes Regulated by Bezafibrate Exhibited Significant Anti‐Tumor Effects

**Figure 6:**
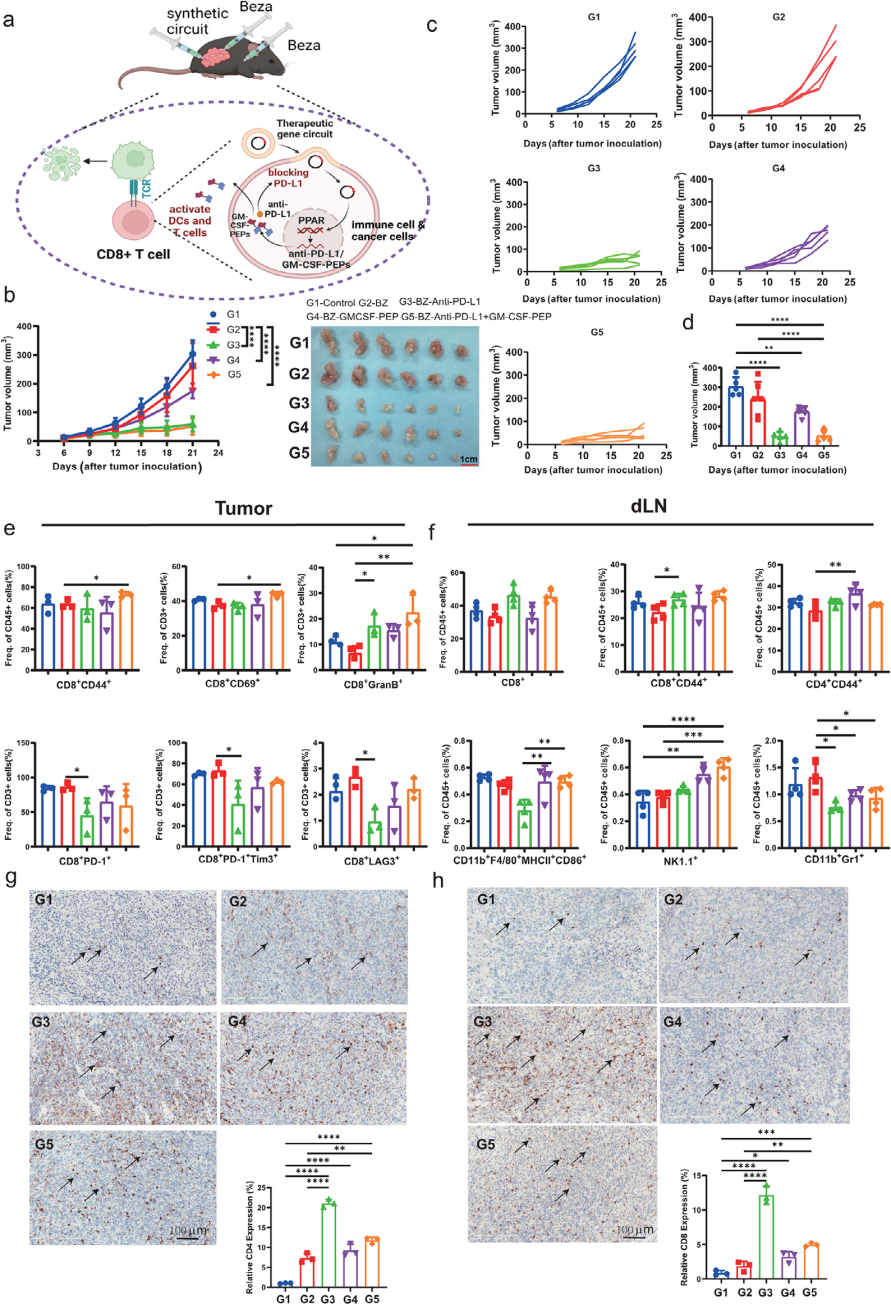
In situ injection of the bezafibrate‐regulated synthetic gene circuit to inhibit bladder cancer growth. a) Schematic illustration of plasmids encoding synthetic therapeutic genes induced anti‐tumor responses via in situ injection. b) In the therapeutic mouse model, Vaccination of mice with plasmids expressing synthetic genes led to the rejection of the cancer cells within 3 weeks and a significant overall reduction in MB49 tumor size (n = 6 per group); Quantification of the tumor size data presented in (b) and Representative images shown in (c). d) The tumor size was on the last day for each group (n = 5). e,f) Quantitative analysis of different subsets of immune cells in TILs (n = 3) (e), and lymph nodes (n = 4) (f) by flow cytometry. g,h) Immunohistochemical staining results of CD4^+^ T cells (14‐0042‐82, Invitrogen) or CD8+ T cells (14‐0081‐82, Invitrogen) in a mouse tumor. The image displayed represents one mouse in each group, with 20× magnification. Positive cell ratio of CD4+ T or CD8+ T cells. Statistical analysis employed a one‐way ANOVA or two‐way ANOVA followed by Tukey's post hoc test (b, c). or an unpaired, two‐tailed Student's t‐test, as appropriate.*, p < 0.05; **, p < 0.01; ***, p < 0.001; ****, p < *0.0001*.

Intratumoral injection of cancer vaccines represents another effective approach to treating tumors. To investigate whether the synthetic circuit can directly inhibit tumor growth, we used in vivo transfection reagents to deliver Gal4‐RXR/PPAR‐p65‐UAS plasmids into the tumor. We tested the efficiency of intratumoral transfection in two tumor models, MB49 and LLC. The target gene was effectively expressed in both models without leakage into surrounding tissues (such as lymph nodes and subcutaneous fat) (Figure S4, Supporting Information). Concurrently, bezafibrate was injected intratumorally to regulate the expression of target genes. Notably, GM‐CSF and peptides secreted by cells serve as potent tumor vaccines. Conversely, PD‐L1 nb cannot be secreted by cells; instead, they are expressed within immune cells or tumor cells to reduce PD‐L1 expression (**Figure** **6**a).

The results demonstrated that the groups injected with bezafibrate combined with PD‐L1 nb plasmids, as well as the group injected with bezafibrate combined with both PD‐L1 nb and GM‐CSF‐peptides, significantly inhibited tumor growth (Figure 6b,c). The group injected with bezafibrate combined with GM‐CSF‐peptides showed a slight reduction in tumor growth (Figure 6b,c). This result was further confirmed by the tumor volume on the final day (Figure 6d). Additionally, the administration of GM‐CSF‐peptides plasmids promoted the expansion of specific T cells upon stimulation with ANLN or CCNB1, but not with CLDN6. However, co‐administration with PD‐L1 nb plasmids induced the expansion of T cells upon stimulation with both ANLN and CLDN6 (Figure S5a, Supporting Information).

We measured the proportions of various immune cell subsets to determine the potential impact of PD‐L1 nb and GM‐CSF‐peptides on the antitumor response. The results revealed that the GM‐CSF‐peptides group exhibited increased DC and macrophage subsets in the blood (Figure S4b, Supporting Information). CD8^+^ memory T cells did not change in any group in the blood (Figure S5b, Supporting Information). In dLNs, CD8^+^ memory cells were elevated in the PD‐L1 nb and co‐transfected groups, whereas CD4^+^ memory cells were only increased in the GM‐CSF‐peptides group. However, NK cells showed an increase in the GM‐CSF‐peptides and co‐transfected groups. Notably, myeloid‐derived suppressor cells (MDSCs) were decreased in all the transfected groups (Figure 6f)

In the tumor microenvironment, administration of PD‐L1 nb plasmids reduced PD‐1 expression on CD8^+^ T cells and mitigated T cell exhaustion, while co‐administration with GM‐CSF‐peptides significantly increased memory CD8^+^ T cells and activated CD8^+^ T cells (CD8^+^CD69^+^). Additionally, all three transfected groups exhibited an increase in effective CD8^+^ T cells (CD8^+^GranB^+^) (Figure 6e). However, GM‐CSF‐peptide‐treated alone failed to elicit strong immunoregulatory effects, aligning with observed tumor growth patterns. Consistent with these findings, intratumoral CD4^+^ and CD8^+^ T cells were more highly infiltrated in the PD‐L1 nb and co‐treatment groups compared to the GM‐CSF peptide‐only group (Figure 6g,h). Collectively, these results indicate that intratumoral expression of PD‐L1 nb effectively inhibits tumor progression and alleviates T cell exhaustion. Furthermore, co‐expression with GM‐CSF‐peptides augments CD8^+^ T cell activity and potentiates the functionality of effector cytotoxic T cells.

### 2.7 Validation of Anti‐Tumor Effects of Bezafibrate‐Regulated PD‐L1 nb Expression

**Figure 7:**
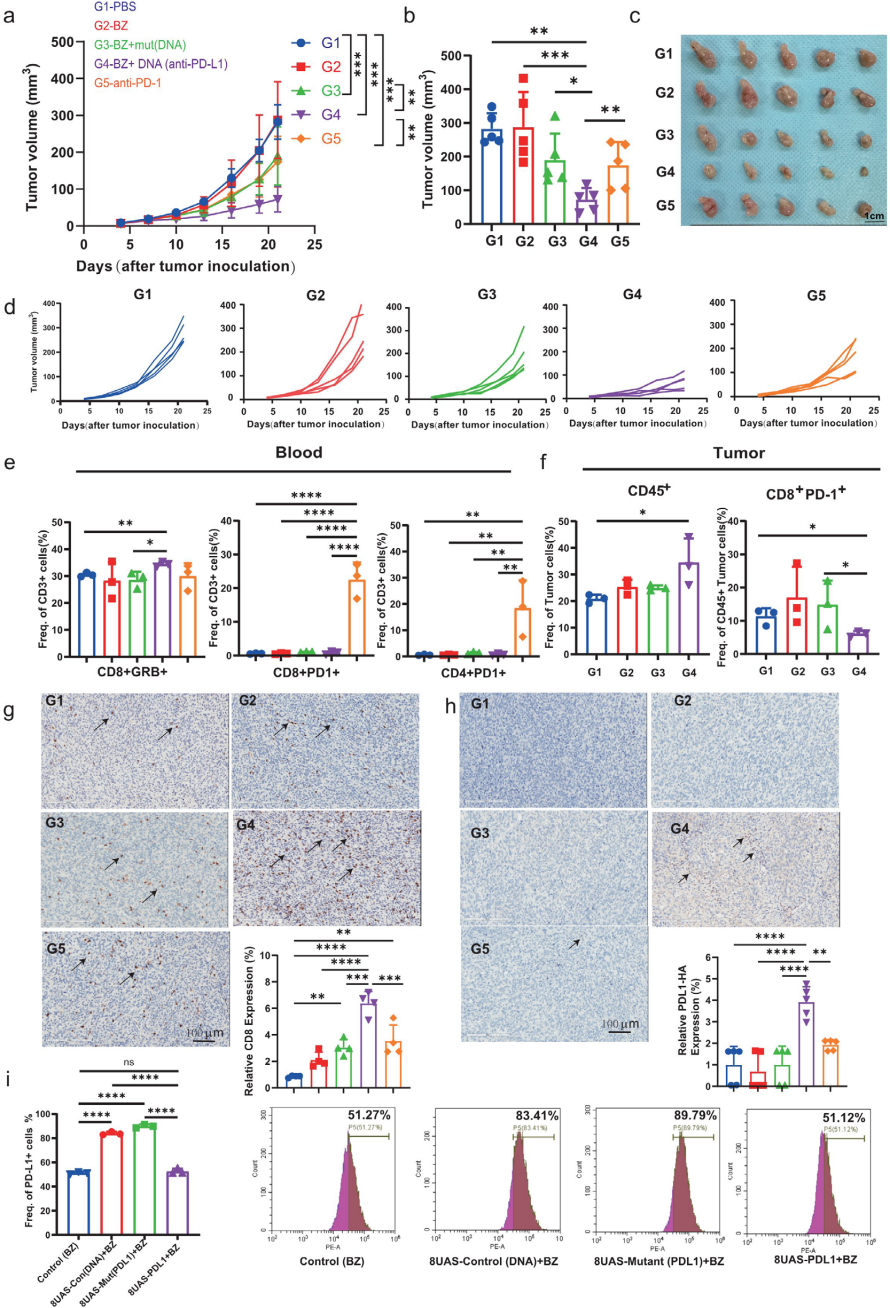
Validation of the anti‐tumor effects of bezafibrate‐regulated synthetic gene circuit. a) Therapeutic mouse model assessing the impact of vaccination with plasmids expressing synthetic and mutated genes on tumor growth (n = 5 per group). Quantifying tumor size data is presented in (a), with representative images in (c). b). Quantification of tumor size was on the final day of the experiment for each group (n = 5). d) Tumor growth curves for each group, illustrating the therapeutic efficacy over time. e) Quantitative analysis of different subsets of immune cells in PBMCs by flow cytometry (n = 3). f) On day 0, C57BL/6J mice were subcutaneously (s.c.) injected with 3×10^5^ MB49 cells in the right flank. Once the tumors reached 80 mm^3^, synthetic gene circuits encoding target genes or mutated genes were injected into the tumors, accompanied by bezafibrate administration. After 40 h, the proportions of CD45^+^ and CD8^+^PD‐1^+^ cells in the tumors were analyzed by flow cytometry. g,h) Immunohistochemical staining results of CD8^+^ T cells or expression of HA tag (PD‐L1 nb contain HA tag) were stained with CD8^+^ specific antibody (14‐0081‐82, Invitrogen) and HA‐specific antibody (3724T, Cell signaling) in mouse tumor. The image displayed represents one mouse in each group, with 20× magnification. Positive cell ratio of CD8^+^ T or expression of PD‐L1nb. i) MB49 cells were treated with bezafibrate, or transfected with synthetic gene circuit plasmids expressing 8UAS‐control DNA (EGFP), 8UAS‐mutant PD‐L1 nb (mutant ATG to TAG), or 8UAS‐PD‐L1 nb and simultaneously treated with 100 µm bezafibrate. After 24 hours, the expression of cell surface PD‐L1 was analyzed by flow cytometry. Data are shown as mean ± SD. One‐way ANOVA or two‐way ANOVA followed by Tukey's post hoc test or unpaired two‐tailed Student's t‐test. *, p < 0.05; **, p < 0.01; ***, p < 0.001; ****, p < *0.0001*.

To validate the anti‐tumor effects of the bezafibrate‐controlled gene regulation system in vivo, we mutated the start codon (ATG) to a stop codon (TAG) in the PD‐L1 nb sequence and introduced the synthetic genes into tumors. To compare the anti‐tumor effects of PD‐L1 nb with those of anti‐PD‐1 antibodies, we also administered anti‐PD‐1 antibodies to the tumors. The results showed that bezafibrate‐regulated target gene expression significantly inhibited tumor growth compared to the mutant group and the anti‐PD‐1 antibody‐treated groups (**Figure** **7**a–d). Notably, the synthetic gene circuit group augmented the proportion of active CD8^+^ T cells (CD8^+^GranB^+^) in blood. However, anti‐PD‐1 antibody injection upregulated PD‐1 expression on both CD8+ and CD4^+^ T cells in the blood (Figure 7e), increasing the side effects associated with anti‐PD‐1 therapy. Additionally, PD‐L1 nb expression promoted greater infiltration of CD8^+^ T cells into tumors compared to the other groups (Figure 7g).

We further assessed the distribution of PD‐L1 nb in tumors by detecting the HA tag integrated into the PD‐L1 sequence. The results showed that while the mutant group lacked HA expression, the synthetic gene construct group exhibited robust HA expression within the tumors (Figure 7h). These findings confirm that PD‐L1 nb expression enhances the proportion of CD8^+^ T cells and inhibits tumor growth. Next, we analyzed the expression of the target and mutant genes regulated by bezafibrate within the tumors. The expression of the target gene directly suppressed PD‐L1, thereby reducing the CD8^+^PD‐1^+^ population, consistent with previous research, ^[^ ^21^ ^]^ whereas the mutant PD‐L1 nb failed to do so (Figure 7f). Finally, we transfected synthetic gene circuits (including the control DNA, mutant PD‐L1 nb, and PD‐L1 nb) into MB49 cells for 24 hours, followed by an analysis of PD‐L1‐positive MB49 cells (Figure 7i). Bezafibrate‐regulated PD‐L1 nb expression significantly reduced PD‐L1 levels in MB49 cells compared to other treatments. These data confirm the efficacy of bezafibrate‐regulated PD‐L1 nb expression both in vitro and in vivo, leading to reduced PD‐L1 expression in tumor cells and contributing to the inhibition of tumor growth.

## 3 Discussion

Genetically engineered cancer vaccines can introduce a wide array of known and novel antigens to the host immune system, thereby facilitating the development of effective tumor vaccines.^[^ ^22^ ^]^ Synthetic molecular switches that enable precise control of gene expression in mammalian cells or tissues in response to safe drugs serve as invaluable tools for elucidating the function of genes of interest and for developing safer, more effective gene and engineered cell therapies.^[^ ^23^ ^]^ In this study, we focused on the clinically relevant and bioactive inducer bezafibrate, developing a bezafibrate‐dependent synthetic gene circuit that demonstrates robust performance in engineered human cells and embryonic stem cells both in vitro and in vivo.

Analogous to classical ON‐type systems, PPAR interacts with RXRα and binds to its cognate DNA‐binding sequence, PPREs, in selected genes such as ACOA, HMG, HMG‐FABP, and IDEAL, activating downstream gene transcription in the presence of bezafibrate. While natural ON‐type switches are easily used for in vitro applications, PPREs often display low promoter activation levels. To address this, we devised a second switch configuration exhibiting ON‐type behavior, wherein bezafibrate‐inducible dimerization of two distinct PPAR LBD domains of a split transcription factor, Gal4 DBD and RXRα fused to a transactivation domain (VPR, VP65, or p65), come together to activate transgene expression in the presence of bezafibrate.

Through functional phenotypic screening, we identified pairs of AD variants that exhibited minimal signal output in the absence of bezafibrate and high activation in its presence, surpassing the gene expression fold changes achieved with natural PPREs. The optimal switch construct, incorporating a potent promoter and an optimal number of UAS elements, responded sensitively to bezafibrate. Our systematic screening revealed that bezafibrate effectively modulates the combination of p65 AD, the CMV promoter, and eight UAS elements, functioning as an efficient switch for target gene expression.

Furthermore, we integrated therapeutic genes, including GM‐CSF, tumor epitopes derived from ESCs, and PD‐L1 nb, into our synthetic gene switch. In vitro experiments demonstrated minimal expression of these genes in the absence of bezafibrate, with significant upregulation upon its addition. This conditional expression preserved GM‐CSF functionality while facilitating the co‐secretion of antigenic peptides, creating a dual‐functional tumor vaccine. The secretion of GM‐CSF‐fused peptides in HEK 293T cells validated our system's efficacy in generating potent immunogens.

In vivo experiments showed that bezafibrate stimulated engineered cell implants microencapsulated in alginate beads, engineered ESCs, or plasmid transfection in tumors, eliciting the expression of the output signal. Administration of bezafibrate every three days in mice triggered the release of sufficient GM‐CSF and tumor antigens or PD‐L1 nb, effectively repressing tumor growth and inducing upregulation of anti‐tumor immune cells in the blood, dLN, and tumor, respectively. In engineered cells encapsulated in alginate beads and engineered ESC models, bezafibrate induced an increase in specific T cells in response to NUf2 and MB49 in the spleen or tumors. The advantages of bezafibrate as an inducer for controlling gene‐ and engineered cell‐based therapies include its excellent safety profile, owing to its widespread use in various diseases ^[^ ^24^ ^]^ and cancer therapy.^[^ ^25^ ^]^ Notably, the synergistic effect of bezafibrate and anti‐PD‐1 antibodies shows enhanced tumor‐killing efficacy, which has already been demonstrated in clinical studies.^[^ ^20^ ^]^

Even though many chemical and synthetic biology approaches have been developed and applied to treat a variety of cancer types, only a few cancer vaccines have been approved by the U.S. Food and Drug Administration (FDA) over the past few decades, including sipuleucel‐T (Provenge), T‐VEC (Imlygic), Cervarix, and Gardasil [33, 34]. A pivotal constraint hindering the efficacy of cancer vaccines lies in their inefficient in vivo delivery and associated side effects, where administered vaccines frequently fail to reach their intended targets.^[^ ^26^ ^]^ In our study, we utilized bezafibrate to regulate PD‐L1 nb expression in tumor cells, significantly reducing PD‐L1 expression in vitro. Induced high expression of PD‐L1 nb in ESCs and tumors enhanced safety and efficiency while limiting secretion, thereby improving the tumor microenvironment and the effectiveness of the tumor vaccine compared to treatments with anti‐PD‐1 antibodies. This was confirmed using mutant PD‐L1nb. In ESC and intratumoral models, PD‐L1 nb delivered and expressed in ESCs or immune/tumor cells effectively mitigated the exhaustion of CD8+ T cells and concurrently increased the proportion of active and effective CD8+ T cells, enabling them to effectively kill tumor cells.

In contrast, GM‐CSF‐peptides secreted within ESCs or the tumor exhibited modest tumor inhibitory effects, potentially due to the immunosuppressive microenvironment. However, co‐transfection with PD‐L1 nb resulted in a significant increase in active and specific T cells within tumors (Figures 5e and 6g). Although engineered PD‐L1 nb expressed in bacteria has been shown to inhibit tumor growth, it typically requires a combination with CTLA4 nb and GM‐CSF.^[^ ^7^ ^]^ In our system, engineered PD‐L1 nb alone significantly suppressed tumor growth.

In summary, by utilizing bladder tumors as our disease model and employing GM‐CSF‐peptide and PD‐L1 nb as the therapeutic outputs, the bezafibrate‐regulated genetic switch demonstrates remarkable adaptability for controlling the in situ production of diverse therapeutic proteins aimed at cancer treatment. Notably, the expression of PD‐L1 nb significantly inhibited bladder tumor growth by mitigating T cell exhaustion and enhancing the activation of effective T cells, thereby amplifying the antitumor effects elicited by tumor antigens derived from ESCs.

Furthermore, these findings underscore the promising potential of the genetic switch and ESC‐derived tumor antigens combined with PD‐L1 nb as an innovative therapeutic approach for bladder tumors. This strategy highlights a new direction in cancer gene therapy, leveraging the safety and efficiency of bezafibrate regulation to enhance immune responses and improve clinical outcomes in cancer treatment.

## 4 Experimental Section

### Materials

Peptides were synthesized by GL Biochem (Shanghai, China). Dulbecco's Modified Eagle (DMEM) Medium, Roswell Park Memorial Institute (RPMI) 1640 medium, fetal bovine serum (FBS), and penicillin‐streptomycin (PS) were obtained from ThermoFisher Scientific (Waltham, MA, USA). Enzyme‐linked immunospot (ELISPOT) assay kit was purchased from MabTech (Nacka Strand, Sweden). Bezabifrate was purchased from MCE (MedChemExpress, USA). Please refer to **Table** **1** for other reagents and antibodies.

**Table 1:** Antibodies and materials.

	Reagent or resource antibody	Source	Identifier
1	PE/Cy7 anti‐mouse CD3	Biolegend	100220
2	APC anti‐mouse CD4	Biolegend	100412
3	Alexa Fluor 700 anti‐mouse CD25	Biolegend	102024
4	PerCP/Cyanine5.5 anti‐mouse CD8a	Biolegend	100733
5	Pacific Blue anti‐mouse/human CD44	Biolegend	103020
6	Brilliant Violet 605 anti‐mouse CD45	Biolegend	103139
7	PE/Dazzle 594 anti‐human/mouse Granzyme B Recombinant	Biolegend	372207
8	Alexa Fluor 488 anti‐mouse FOXP3	Biolegend	126405
9	PE anti‐mouse H‐2Dk	Biolegend	110307
10	Brilliant Violet 421 anti‐mouse F4/80	Biolegend	123131
11	PE/Dazzle 594 anti‐mouse I‐A/I‐E	Biolegend	107647
12	FITC anti‐mouse CD86	Biolegend	105005
13	PE/Cy7 anti‐mouse/human CD11b	Biolegend	101215
14	APC anti‐mouse CD11c	Biolegend	117309
15	PerCP/Cyanine5.5 anti‐mouse CD49b (pan‐NK cells)	Biolegend	108915
16	Alexa Fluor 700 anti‐mouse Ly‐6G/Ly‐6C (Gr‐1)	Biolegend	108421
17	PE/Cy7 Rat IgG2b, κ Isotype Ctrl	Biolegend	400617
18	APC Rat IgG2b, κ Isotype Ctrl	Biolegend	400611
19	PerCP/Cyanine5.5 Rat IgG2a, κ Isotype Ctrl	Biolegend	400531
20	Pacific Blue Rat IgG2b, κ Isotype Ctrl	Biolegend	400627
21	Brilliant Violet 605 Rat IgG2b, κ Isotype Ctrl	Biolegend	400657
22	Alexa Fluor 488 Rat IgG2b, κ Isotype Ctrl	Biolegend	400625
23	PE Mouse IgG2a, κ Isotype Ctrl	Biolegend	400211
24	Brilliant Violet 421 Rat IgG2a, κ Isotype Ctrl	Biolegend	400549
25	PE/Dazzle 594 Rat IgG2b, κ Isotype Ctrl	Biolegend	400659
26	FITC Rat IgG2a, κ Isotype Ctrl	Biolegend	400505
27	Alexa Fluor 700 Rat IgG2b, κ Isotype Ctrl	Biolegend	400628
28	CD4 Monoclonal Antibody (RM4‐5), eBioscience	Invitrogen	14‐0042‐82
29	CD8a Monoclonal Antibody (53‐6.7), eBioscience	Invitrogen	14‐0081‐82
30	HA‐Tag (C29F4) Rabbit mAb #3724	Cell signaling	3724
31	TruStain fcX (anti‐mouse CD16/32)	Biolegend	101320
32	True‐Nuclear Transcription Factor Buffer Set	Biolegend	424401

	Chemicals, peptides and Recombinant Proteins		
1	Penicillin‐Streptomycin‐Glutamine (100X)	Gibco	10378016
2	FBS	Gibco	10099‐141
5	DMEM	Gibco	10566‐016
6	Opti‐MEM	Gibco	31985070
9	Trypsin‐EDTA (0.25%), phenol red	Gibco	25200072
10	InVivoMab anti‐mouse CD4	BioXcell	BE0003‐1‐5MG
11	InVivoMab anti‐mouse CD8	BioXcell	BE0004‐1‐5MG
13	100 µm cell strainer	Falcon	352360
14	70 µm cell strainer	Falcon	352350
15	Percoll	GE Health	17‐0891‐02
16	DPBS	Hyclone	SH30028.02
22	TLRL‐1826 ODN	Nidogen	tlrl‐1826‐1
24	Dimethyl sulfoxide (DMSO)	Sigma–Aldrich	D2650
25	Collagenase A	Roche	10103586001
27	HBSS	Servicebio	G4204‐500
28	10XPBS	Servicebio	G4207‐500
32	mouse IFN‐gamma elispot plus(plus) HRP	MabTech	3321‐4HST‐2
34	Bovine Serum Albumin	SIGMA	V900933‐100G
36	mouse IFN‐gamma elispot plus(plus) HRP	MabTech	3321‐4HST‐2
37	CD45 MicroBeads, mouse	Miltenyi Biotec	130‐052‐301
38	2’3’‐cGAMP VacciGrade	Invivogen	
39	Mouse IFN‐gamma ELISpot PLUS (HRP), strips	MabTech	3321‐4HST‐10
40	EmbryoMax Nucleosides (100X)	Merck	ES‐008

### Cell Culture and Transfection

Mouse ESC (129) were gifts from Prof. Pentao Liu of the University of Hong Kong. The ESCs were cultured in Knockout DMEM (ThermoFisher Scientific) supplemented with 15% (vol/vol) FBS, 1× (vol/vol) MEM NEAAs (Stemcell, BC, Canada), 1× (vol/vol) glutamine–penicillin‐streptomycin (ThermoFisher Scientific), 0.1 µM m 2‐mercaptoethanol, and 10^3^ U mL^−1^ human LIF. The ESCs and iPSCs were grown for several passages and transferred to 0.1% gelatin‐coated plates for SSEA‐1 magnetic bead sorting (Miltenyi, Germany).

The MB49 bladder cancer cell line (SCC148, Sigma–Aldrich, St. Louis, MO, USA) was grown in DMEM with 10% FBS and 1% PS in a humidified atmosphere at 37 °C and 5% CO_2_. Human embryonic kidney cells (HEK‐293T; ATCC), were cultured in DMEM (Invitrogen) supplemented with 10% FBS and 1% PS in a humidified atmosphere at 37 °C and 5% CO_2_ (Invitrogen). HEK‐293T cells were cultivated in cotransfection, a 5:1 or 10:1 receptor/reporter plasmid ratio was used) diluted in 1 µL P3000 and 25 µL Opti‐MEM solution, subsequently mixed with 25 µL of Opti‐MEM solution and 1.5 µL lipofectamine 3000 (Invitrogen). The DNA‐containing solution was added dropwise to the cells and the medium was replaced after 6 h.

### Plasmid Construction

All plasmid constructs were generated using homologous recombination methods. PCR amplification was performed using Takara PrimerSTAR Max DNA Polymerase (R045, Takara, 2×Mix), and subsequent ligation reactions were conducted with the ClonExpress Ultra One Step Cloning Kit V1/V2 (C115/C116, Vazyme). Trans5α Chemically Competent Cells (CD201, Transgen) were used as the transformation strain. The pGL3‐Basic vector without a promoter (VT1554, YouBio) encoding luciferase and the pRL‐TK vector (VT1568, YouBio) encoding Renilla luciferase were utilized in luciferase assays to test the transcription factor binding capacity. For transient expression, vectors such as pcDNA3.1, pcDNA3.1‐EGFP, and pEGFP/mCherry‐N1 were employed for the construction of activators and subsequent in vivo experiments. The target fragments, PPARγ LBD (UniProt: P37238, aa238‐aa503), RXRα LBD (UniProt: P28700, aa232‐aa463), Gal4 DBD, and p65 AD were synthesized and assembled by BGI. The VP64 domain was cloned from the plasmid pHR_PGK_antiCD19_synNotch_Gal4VP64 (P0920, Miaoling Biology), and the VP64‐p65‐Rta (VPR) fragment was cloned from SP‐dCas9‐VPR (P1290, Miaoling Biology) via PCR and homologous recombination into the pmCherry‐N1 vector, fused with Gal4 DBD separately in a single expression cassette. The pre‐screened neoantigen‐encoded sequences used for therapeutic purposes, along with the adjuvant GM‐CSF (UniProt: P01587)‐encoded sequence, were synthesized and assembled by BGI. These synthetic fragments were subsequently subcloned to the expression vectors in our laboratory. All recombinant plasmids were sequence‐verified by Sangon Biotech before in vitro and animal experiments. Please refer to the plasmid and primer list (Tables S1 and S2, Supporting Information) for further details.

### Reporter Gene Activity Assays

HEK 293T cells were plated in white 96‐well plates (30 196, SPL) at a density of 1.5×10^4^ cells per well. The following day, transfection was performed by mixing 0.1 µg of total plasmid DNA in a ratio of UAS‐P_core_: Gal4DBD‐RXRαLBD: PPARγLBD‐p65AD (90:5:5) with 0.2 µL of Hieff Trans Liposomal Transfection Reagent (40802ES, YEASEN) in 50 µL of Opti‐MEM (31 985 070, Gibco). Cells were incubated at 37 °C in 5% CO₂ for 6 hours before replacing the medium with DMEM supplemented with 10% FBS and varying concentrations of bezafibrate. Luciferase activity was assessed at 24 or 48 h post‐treatment using the Dual‐Luciferase Reporter Gene Assay Kit (RG028, Beyotime) according to the manufacturer's instructions. To avoid bias caused by different reagents, the Duo‐Lite Luciferase Assay System (DD1205, Vazyme) was also used to perform luciferase assays in different batches.

### Fluorescence Imaging

EGFP expression was visualized using a Nikon Confocal laser scanning microscope (Confocal laser scanning microscope, Nikon, A1R) equipped with a Nikon digital camera, a 10×objective, a 488‐nm/405‐nm/561‐nm (B/G/R) excitation/emission filter set, and Image‐Pro Express C software (versionipp6.0).

### Western Blotting

The protein expression levels of released GM‐CSF were verified by Western blotting. Supernatant samples were treated with RIPA lysis buffer. A BCA assay kit (Thermofisher, USA) was used to determine the protein concentration. An equal amount of protein (20 µg) was loaded into lanes. After being separated by electrophoresis, proteins were electrically transferred to a PVDF membrane (Millipore, USA). After being blockaded with 5% milk, the membrane was incubated with the primary antibodies: anti‐GM‐CSF (Sigma–Aldrich, St. Louis, MO, USA), and anti‐β‐actin (Abcam, Cambridge, MA, USA), each was diluted at a ratio of 1:1000 and incubated over‐night at 4 °C. After incubation with the corresponding horseradish peroxidase‐linked secondary antibody at room temperature for 1 h, target proteins were developed by the enhanced chemiluminescence kit (Millipore, USA).

### Animal Experiments

C57BL/6J mice (6 weeks old, male) weighing ≈25 g, were used. Animals were housed in a controlled room at 22 °C, 50% humidity, 12‐h light‐dark cycle with adlibitum access to standard diet and drinking water. Animals were randomly assigned to experimental groups. *Cell implants*. Engineered HEK‐293T cells were encapsulated into coherent alginate‐poly‐(L‐lysine)‐alginate beads (240 µm; 200 cells /capsule) using an Inotech Encapsulator Research Unit IE‐50R (Buechi Labortechnik AG) with the following parameters: 10 mL syringe operated at a flow rate of 400 units, 120 µm nozzle with a vibration frequency of 1000 Hz, and bead dispersion voltage of 1.5 kV.

The MB49 bladder cancer cell line (SCC148, Sigma–Aldrich, St. Louis, MO, USA) is syngeneic to C57BL/6 mice and has low‐grade lymphoid metastatic potential for the lungs. Encapsulated cells were injected subcutaneously (s.c.) into the right flank of male C57BL/6 J mice using a syringe every week (Terumo). Three weeks later, 5×10^4^ cancer cells were resuspended in 100 µL PBS and injected subcutaneously in the lower back of the C57BL/6 J mice to generate the prophylactic model. Body weight and overall appearance were monitored weekly to detect early signs of autoreactivity to the vaccine. Tumor growth was measured by digital calipers every 3 days. On day 5 after the last immunization, mice were sacrificed and blood, tumor, spleen, and draining lymph nodes (dLNs) were harvested. Blood samples were collected in sterile 1.5 mL microtubes and anticoagulant tubes to obtain serum and plasma, respectively. Animal protocols were approved by the Institutional Animal Care and Use Committee (IACUC), Shenzhen Institute of Advanced Technology (SIAT), Chinese Academy of Science.

### Injection with Engineered ESC Vaccines

Male C57BL/6J mice were subcutaneously injected with 4×10^5^ MB49 bladder cancer cells in the right flank. The success rate of MB49 tumor implantation in mice was ≈90%. To utilize ESCs for the delivery of synthetic gene circuit, several plasmids were transiently transfected into the ESCs. 2×10^7^ ESCs were cultured in T75 flanks, plasmids for Gal4‐RXR, PPAR‐p65, 8UAS‐CMV‐GM‐CSF‐PEPs or/and 8UAS‐CMV‐ PD‐L1 nb transfected into ESCs using Xfect mESC Transfection Reagent (Clontech) according to the manufacturer's instructions. For the ESC‐based vaccine, 2×10^6^ SSEA‐1‐sorted syngeneic murine ESCs were irradiated at 6000 rads before injection. Cells were suspended in 100 µL of 5 µ m CpG (Invivogen, San Diego, USA) in PBS. Mice were placed in an induction chamber and anesthetized with 2% isoflurane (Isothesia, Butler Schein) in 100% oxygen at a delivery rate of 2 L min^−1^ until loss of righting reflex, according to the IACUC guidelines at SIAT. ESCs were injected subcutaneously (s.c.) into the right flank of male C57BL/6J mice. Body weight and overall appearance were monitored weekly to detect early signs of autoreactivity to the vaccine. The same vaccine dosage and preparation were used for the therapeutic, and adjuvant treatments. Tumor growth was measured by digital calipers every 3 days.

### Tumor In Situ Injection of DNA Plasmids

MB49 tumor model was established as described above. When the tumor volume reached about 80 mm^3^, mice were divided into five groups including PBS, bezafibrate, bezafibrate+ PD‐L1 nb (10 µg/each mice, plasmid), bezafibrate + GM‐CSF‐peptides (10 µg/each mice, plasmid), bezafibrate+ PD‐L1 nb (5 µg/each mice, plasmid) + GMCSF‐peptides (5 µg/each mice, plasmid). To perform the genetic circuit expression in the tumor, 10 µg of plasmid DNA for Gal4‐RXRa, PPAR‐p65, 8UAS‐CMV‐GM‐CSF‐PEPs or/and 8 UAS‐CMV‐ PD‐L1 nb, were complexed in vivo JetPEI (catalog no. 101 000 040, Polyplus, New York, USA) with or without injected 100 µ m bezafibrate together every three days. The tumor size of each group was recorded every 3 days. Tumor volume was calculated according to the formula: width^2^ × length/2. After 21 days of monitoring, tumor tissues were collected by flow cytometry analysis.

To evaluate the mechanisms by which bezafibrate modulates the gene circuit and target gene expression, the start codon was mutated to a stop codon within the PD‐L1 nb sequence, subsequently examining the in vivo regulation of the gene circuit by bezafibrate. Male C57BL/6J mice were subcutaneously inoculated into the right flank with 3 × 10^5^ MB49 bladder cancer cells. Once the tumor volume attained ≈80 mm^3^, the mice were stratified into five groups: 1) PBS control, 2) bezafibrate treatment, 3) bezafibrate combined with mutant PD‐L1 nb (10 µg/mouse, plasmid‐based), 4) bezafibrate combined with wild‐type PD‐L1 nb (10 µg/mouse, plasmid‐based), and 5) anti‐PD‐1 antibody treatment. The tumor size of each group was meticulously recorded every 3 days.

### Isolation of Lymphocytes from Spleens, draining lymph nodes (dLNs), and Tumors

Spleens were gently mashed using a 2‐mL syringe plunger, filtered through a 70‐µm strainer (Falcon, Corning, Germany), and then suspended in 1640 medium, followed by centrifugation to obtain splenocytes. After adding erythrocyte lysis buffer and washing in PBS, cells were collected for ELISPOT assay or stored at −80 °C until further use. The dLNs were processed as described above to acquire lymphocytes. Tumor tissues were ground by a pestle and cultured in RPMI medium with FBS, collagenase, DNase, and 4‐(2‐hydroxyethyl)‐1‐piperazineethanesulfonic acid (HEPES). After shaking at 37 °C for 20 min, the samples were filtered through a 100‐µm strainer and suspended in percoll gradient (General Electric Healthcare, USA) to remove non‐immune cells. Next, ammonium chloride potassium (ACK) lysis buffer was used to remove red blood cells. After washing with PBS, tumor‐infiltrating leukocytes (TILs) were collected for ELISPOT assay or stored at −80 °C for subsequent analyses.

### ELISPOT Assay

Lymphocytes derived from spleens or TILs (5 × 10^5^ cells) were co‐cultured with different peptides for 25 h to detect the secretion of IFN‐γ by ELISPOT assay according to the manufacturer's instructions (MabTech, Nacka Strand, Sweden). The size and number of IFN‐γ‐positive spots were calculated using Adobe Photoshop CS6 software.

### Staining of Inflammatory Cells for FACS Analysis

Peripheral blood mononuclear cells (PMBCs) were obtained from blood and suspended in 100 µL FACS buffer containing DPBS and 2% FBS. The PBMCs and lymphocytes derived from dLNs were blocked using an FcR‐blocking Reagent (BioLegend, San Diego, CA, USA) and divided into two groups. One group was stained with a surface marker panel containing CD3, CD4, CD25, CD8, CD44, CD45 (BioLegend), and intracellular markers Granzyme‐B and FoxP3 (BioLegend). The other group was stained for F4/80, MHC‐II, CD86, CD11b, CD11c, NK1.1, Ly6‐G, and CD45 (BioLegend). Rat IgG2b isotype control (BioLegend) was used for CD3, CD11b, CD4, CD11c, CD25, Gr‐1, CD44, CD45, Foxp3, and Granzyme B; rat IgG2a isotype control (BioLegend) for CD86 and F4/80, CD8a and NK1.1; and rat IgG1 isotype control (BioLegend) for MHC II. Staining with intracellular markers required fixation and permeabilization of cells following extracellular staining. Samples were analyzed on a Beckman Flow Cytometer in the Beckmann FACS facility.

### Immunohistochemical Analysis of Tumor

The tumor tissue samples were fixed in 4% paraformaldehyde, embedded in paraffin, and sectioned. The tissue sections were then dewaxed and subjected to antigen retrieval by placing them in citric acid antigen repair solution and heating them in a microwave oven for 10 min. Endogenous peroxidase was blocked by immersing the sections in 3% hydrogen peroxide solution for 10 min, followed by washing with PBS and spin‐drying. The sections were blocked with 3% BSA and then incubated with primary antibody overnight at 4 °C. After that, secondary antibodies corresponding to the species of the primary antibody were incubated for 1 h. The sections were then stained with DAB‐developing solution and hematoxylin to visualize the nuclei. Finally, the sections were dehydrated and observed under a microscope. The positive expression of DAB appeared brownish yellow, while the nuclei stained with hematoxylin appeared blue. Microscopic slides were digitally scanned at 20× magnification using the Zeiss Axioscan 7 (Zeiss, Germany).

### Quantification and Statistical Analyses

All data were analyzed using GraphPad Prism, version10. Normality was assessed using the Shapiro‐Wilk test (for small sample sizes) or the Kolmogorov–Smirnov test (for larger sample sizes). Variance homogeneity was evaluated using Levene's test. For data that met the assumptions of normality and equal variance, parametric tests (e.g., t‐tests or ANOVA) were applied. For multiple comparisons following ANOVA, Tukey's test was applied to control the family‐wise error rate. Statistical significance was set at *p* < 0.05. Differences between tumor growth curves were determined by repeated measures two‐way ANOVA. *∗P <* *0.05, ∗∗P <* *0.01, ∗∗∗P <* *0.001, ∗∗∗∗P <* *0.0001*.

### One Sentence Summary

Bezafibrate regulates the Gal4‐RXRα/PPARγ‐p65/UAS genetic circuit and the expression of GM‐CSF peptides or PD‐L1 nanobodies to inhibit tumor growth.

### Ethics Approval and Consent to Participate

Animal protocols (SIAT‐IACUC‐210202‐HCSJ‐ML‐A1542) were approved by the Institutional Animal Care and Use Committee (IACUC), Shenzhen Institute of Advanced Technology (SIAT), Chinese Academy of Science.

### Consent for Publication

All authors approved the final manuscript and the submission to this journal.

## Conflict of Interest

The authors declare no conflict of interest.

## Supporting information



Supporting Information

Supporting Information

Supporting Information

## Data Availability

The datasets generated during and/or analyzed during the current study are publicly available.
