# Supplementary material for: Engineered Genetic Circuits Activated by Bezafibrate Improve ESC‐Based TAA Cancer Vaccine Efficacy and PD‐L1 Nanobody Therapy
Source: Adv Sci (Weinh). 2025 Apr 17;12(23):2500272. doi: 10.1002/advs.202500272 (PMC12199315; doi:10.1002/advs.202500272)
Supplement: Supplementary file 1 — Supporting Information [file ADVS-12-2500272-s003.docx]

((Supporting Information can be included here using this template))

Supporting Information

Engineered Genetic Circuits Activated by Bezafibrate Improve ESC-Based TAA Cancer Vaccine Efficacy and PD-L1 Nanobody Therapy

Meiling Jin, Shuzhen Liu, Mingshuo Zhan, Jian-Dong Huang ^*^

Meiling Jin, Shuzhen Liu, Mingshuo Zhan

Chinese Academy of Sciences (CAS) Key Laboratory of Quantitative Engineering Biology, Shenzhen Institute of Synthetic Biology, Shenzhen Institutes of Advanced Technology, Chinese Academy of Sciences, Shenzhen, 518055, China.

Jian-Dong Huang

Chinese Academy of Sciences (CAS) Key Laboratory of Quantitative Engineering Biology, Shenzhen Institute of Synthetic Biology, Shenzhen Institutes of Advanced Technology, Chinese Academy of Sciences, Shenzhen, China.

School of Biomedical Sciences, Li Ka Shing Faculty of Medicine, The University of Hong Kong, Pokfulam, Hong Kong SAR, China.

Department of Clinical Oncology, Shenzhen Key Laboratory for cancer metastasis and personalized therapy, The University of Hong Kong-Shenzhen Hospital

Guangdong-Hong Kong Joint Laboratory for RNA Medicine, Sun Yat-Sen University, Guangzhou, China 510120.

Materials Innovation Institute for Life Sciences and Energy (MILES), HKU-SIRI, Shenzhen, P.R. China

Email: jdhuang@hku.hk, Tel: +852 39176810; fax: +852 39176810;

((Please insert your Supporting Information text/figures here. Please note: Supporting Display items, should be referred to as Figure S1, Equation S2, etc., in the main text…)

Experiment section.

*Testing of intratumoral expression of synthetic gene circuits:* Male C57/BL6J mice were subcutaneously injected with 1×10^6^ MB49 bladder cancer cells or 1×10^6^ LLC Lewis Lung Carcinom**a** bladder cancer cellsin the right ﬂank. The success rate of MB49 tumor implantation in mice was approximately 90%. When the tumor volume reached about 50 mm^3^, mice were divided into 2 groups including PBS, bezafibrate+ 8UAS luciferase. To perform the genetic circuit expression in the tumor, 10 μg of plasmid DNA for Gal4-RXRa, PPAR-p65, 8UAS-CMV-luciferase were complexed in vivo JetPEI (catalog no. 101000040, Polyplus, New York, USA) with or without injected 100 μM bezafibrate together. The mice were administered three times in total. Three days after the final injection, they were injected with 75 mg/kg of D-luciferin substrate and observed by an in vivo imaging system for the fluorescence expression in the tumor, lymph nodes, and subcutaneous fat.

Figures Legends


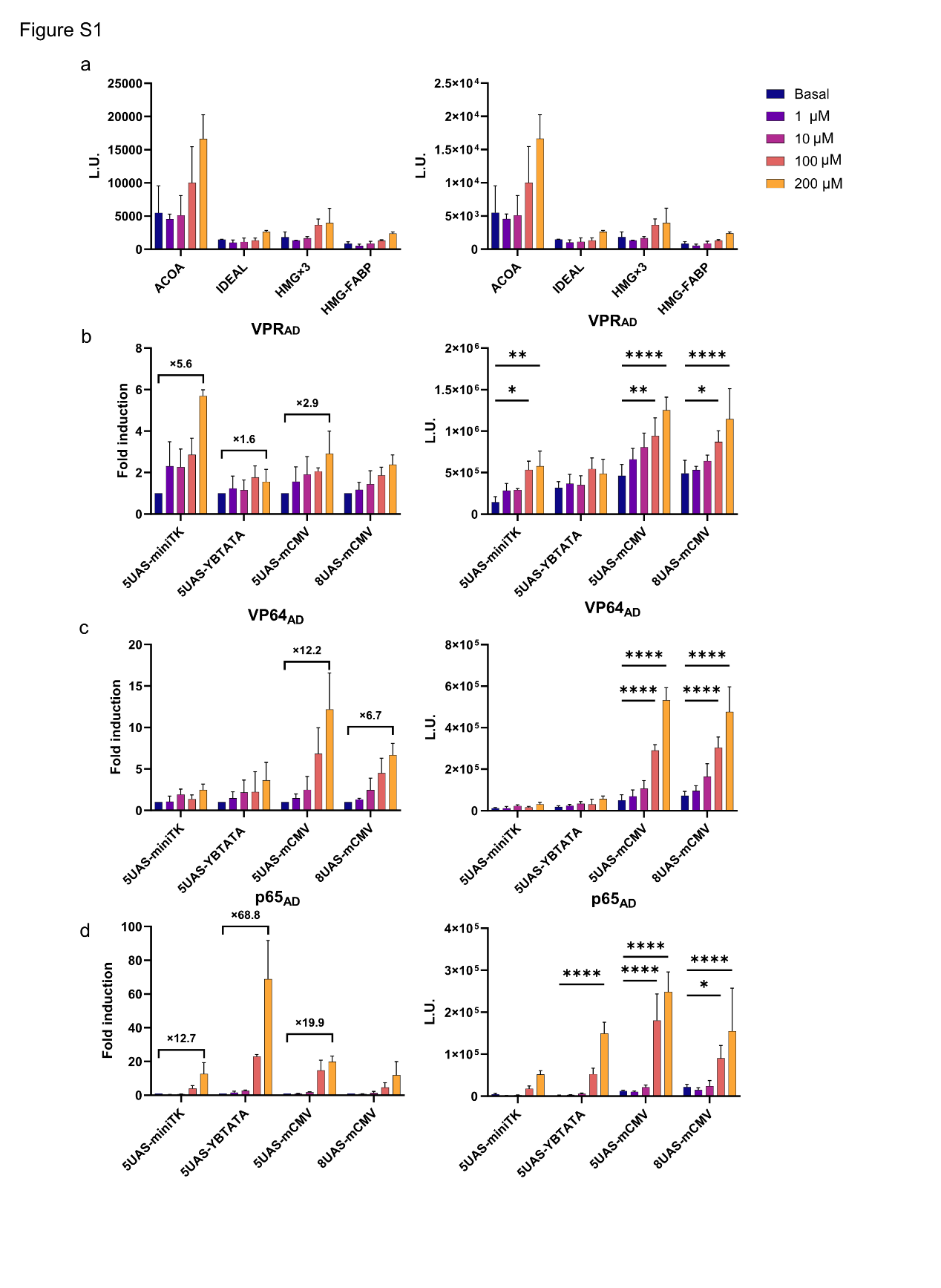


Figure S1. Bezafibrate-induced gene expression control with the synthetic genetic circuit Gal 4-RXRα/PPARγ-VPR/VP64/p65 AD-UAS. Fold-increase of luciferase activity (bezafibrate), and validation of bezafibrate-dependent regulation of the pcDNA 3.1 Gal4 DBD-RXRα LBD, PPARγ LBP-VPR/VP64/p65 AD, 5 x UAS-P_miniTK/ ybTATA/CMV_-luciferase, 8 x UAS-P_CMV_-luciferase constructs for 48 h in transiently transfected HEK293T cells. Data are shown as mean ± SD. e-way ANOVA with a Tukey multiple comparisons test. **, p < 0.05; **, p < 0.01; ***, p < 0.001; ****, p < 0.0001*.


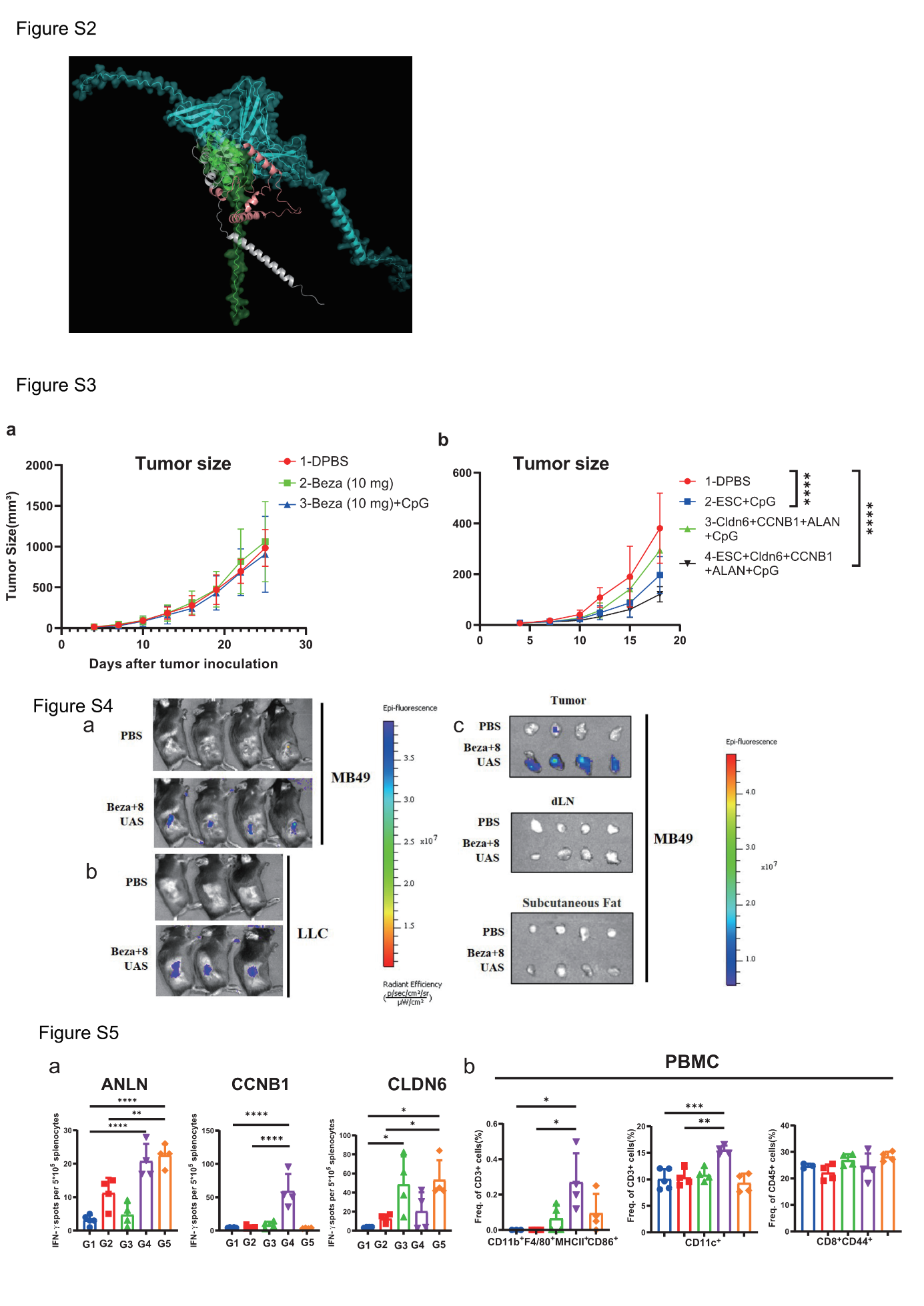


Figure S2. Analysis of GM-CSF-peptides by alphaFold3. The GM-CSF receptor binding domain was analyzed using alphaFold3, which was compared with GM-CSF peptides to assess its impact on GM-CSF binding to its receptor and whether its function is affected. GM-CSF-R is highlighted in cyan, GM-CSF in green, the fused protein (GM-CSF-peptides) with GM-CSF in gray, and EAAAK peptides in salmon.

Figure S3 ESCs combined with peptides inhibit tumor growth. a) C57BL/6 mice were s.c. injected with 4×10^5 MB49 cells in the right flank on day 0, followed by s.c immunized on day 7, 14, 21 for three total vaccinations. Bezafibrate (10mg/kg) with or without CpG (5μm) was administrated i.p. every three days. Anti-tumor effects of bezafibrate with or without CpG were assessed (n = 5 per group). Tumor size was measured every 3 days. b) C57BL/6 mice were s.c. injected with 4×10^5 MB49 cells in the right flank on day 0, followed by s.c immunized on day 7, 14, 21 for three total vaccinations. 2×10^6 ESCs+CpG (5μm), 100 μg of each peptides (Cldn6, Anln, Ccnb1)+CpG (5μm), or ESC + peptides +CpG were administrated every week. Anti-tumor effects of ESCs, peptides, ESC+peptides were assessed (n = 5 per group). Tumor size was measured every 3 days. Two-way ANOVA with a Tukey multiple comparisons test or unpaired two-tailed Student’s t-test. **, p < 0.05; **, p < 0.01; ***, p < 0.001; ****, p < 0.0001.*

Figure S4. Intratumoral expression of synthetic circuits. a, c: C57BL/6 mice were subcutaneously (s.c.) injected with 1×10^6 MB49 cells in the right flank on day 0. When tumors reached approximately 50 mm³, mice were intratumorally injected with 10 µg of DNA plasmid. After 24 hours, D-luciferin was intratumorally injected, and bioluminescence in mice, tumor tissue, lymph nodes, and subcutaneous fat tissue was monitored using a small animal in vivo imaging system (n = 4 per group). b. C57BL/6 mice were subcutaneously (s.c.) injected with 1×10^6 LLC cells in the right flank on day 0. When tumors reached approximately 50 mm³, mice were intratumorally injected with 10 µg of DNA plasmid. After 24 hours, D-luciferin was intratumorally injected, and bioluminescence was monitored using an in vivo imaging system (MB49 model: n=4 per group, LLC model: n = 3 per group).

Figure S5. a) Quantitative analysis of different subsets of immune cells in PBMCs by flow cytometry (n=4). b) Quantitative assessment of IFNγ secretion using ELISPOT assays to detect immune cell activation in TILs against selected epitopes and MB49 tumor cells. G1, PBS. G2, Bezafibrate + CpG, G3, Bezafibrate + CpG + ESCs, G4, Bezafibrate + CpG + ESC (PD-L1nb), G5, Bezafibrate + CpG + ESC (GM-CSF-peptides), G6, Bezafibrate + CpG + ESC (PD-L1nb, GM-CSF-peptides), (n=4). Data are presented as mean ± SD. Statistical significance was determined using one-way ANOVA with a Tukey multiple comparisons test or an unpaired two-tailed Student's t-test. NS, no significance; **, p < 0.05; **, p < 0.01; ***, p < 0.001; ****, p < 0.0001*.
